# Similar revision rates in clinical studies and arthroplasty registers and no bias for developer publications in unicompartmental knee arthroplasty

**DOI:** 10.1007/s00402-020-03336-3

**Published:** 2020-02-08

**Authors:** Georg Hauer, Gerwin A. Bernhardt, Gloria Hohenberger, Lukas Leitner, Paul Ruckenstuhl, Andreas Leithner, Gerald Gruber, Patrick Sadoghi

**Affiliations:** Department of Orthopaedics and Trauma, Medical University of Graz, Auenbruggerplatz 5, 8036 Graz, Austria

**Keywords:** Unicompartmental knee arthroplasty, Revision rate, Arthroplasty register, Systematic review

## Abstract

**Purpose:**

Our aim was to assess the outcome with respect to cumulative revision rates of unicompartmental knee arthroplasty (UKA) by comparing published literature and arthroplasty registry data. Our hypothesis was that there is a superior outcome of UKA described in dependent clinical studies compared to independent studies or arthroplasty registers.

**Methods:**

A systematic review of all clinical studies on UKA in the past decade was conducted with the main endpoint revision rate. Revision rate was calculated as “revision per 100 component years (CY)”. The respective data were analysed with regard to a potential difference of the percentage of performed revision surgeries as described in dependent and independent clinical studies. Clinical data were further compared to arthroplasty registers in a systematic search algorithm.

**Results:**

In total, 48 study cohorts fulfilled our inclusion criteria and revealed 1.11 revisions per 100 CY. This corresponds to a revision rate of 11.1% after 10 years. No deviations with regard to revision rates for UKA among dependent and independent clinical literature were detected. Data from four arthroplasty registers showed lower survival rates after 10 years compared to published literature without being significant.

**Conclusions:**

The outcomes of UKA in dependent and independent clinical studies do not differ significantly and are in line with arthroplasty register datasets. We cannot confirm biased results and the authors recommend the use of UKAs in properly selected patients by experts in their field.

## Introduction

Unicompartmental knee arthroplasty (UKA) is considered a more conservative procedure than total knee arthroplasty (TKA) and provides better physiological function, a less invasive surgical approach, faster recovery time, and shorter hospital stay and rehabilitation [1–5]. UKAs also show a decreased risk of medical complications such as myocardial infarction, venous thromboembolism or deep infection [6].

Despite clinical benefits, however, revision rates are higher compared to TKA [7, 8]. Life expectancy of prostheses is of fundamental importance from a surgeons’ and patients’ perspective and for economic reasons [9–11]. To assess revision rates, data of clinical studies and, on the other hand, national arthroplasty registers can be used. Studies try to extrapolate results of a patient sample to an entire population [10]. On the contrary, registers are designed to comprise all data in a defined region to provide a very realistic picture of surgical outcome data. Therefore, datasets of high-volume registers can be used as a control group when compared to the outcome data of sample-based studies [11].

Regarding the reliability of the data, there is a controversy concerning a potential bias of developer publications compared to register data. Therefore, developer or dependent studies should be examined separately to independent and register studies to assess potential bias. The hypothesis of this study was that there is a superior outcome of UKAs described in dependent clinical studies compared to independent studies or arthroplasty registers.

## Materials and methods

We performed a systematic research using PubMed and Embase with the following search terms: “(arthroplasty, replacement, knee)” AND “((unicompartmental OR unicondylar OR partial) NOT (patellofemoral OR total OR TKA).

Inclusion criteria for consideration in the evaluation were the following: (1) follow-up time had to be 24 months or longer. (2) Revision rates were either mentioned in the text or could be calculated from the available data. Revision surgery was defined as the exchange of at least one component of the prosthesis or conversion to TKA. (3) The used implant must have been clearly specified as UKA and only one single implant or its successor model was used per study cohort. (4) The presented data had to be published in a peer-reviewed journal and to be written in English or German language, and (5) the date of publication was between 2008 and July 2018. To avoid double counting of same study groups in multiple reports, only the study with the longest follow-up period was included. Exclusion criteria were case reports, reviews, former meta-analyses, uncemented fixations techniques and Oxford I/II UKAs.

The following parameters were assessed: title, year of publication, origin of the corresponding author, publishing journal, study design, name of prosthesis, fixation type, treated compartment, number of primary cases, follow-up in years, number of revisions and reason for revision. The systematic analysis was performed according to the PRISMA criteria [12].

Articles were studied in full text and paper were included if they met our inclusion criteria. Finally, we checked the references from included publications for their eligibility to join our study, by hand.

Group comparison was performed between (1) dependent and independent studies and (2) medial and lateral UKA. Studies were rated as *dependent*, if the implant designer was named as an author or co-author, or the developing institution was involved in study design, financial support, or indicated for correspondence. The outcome of single implants was not investigated except for medial Oxford III prostheses.

The National Joint Registers presenting data on UKA were accessed through the EFORT (European Federation of National Associations of Orthopaedics and Traumatology) Website of the Network of Orthopaedic Registries of Europe (NORE) [13]. Data were retrieved concerning 10-year revision rates of UKAs. Four registers were suitable and were used for comparative analysis: the Australian (AOA) [8], the New Zealand (NZJR) [14], the Swedish (SKAR) register [15] and the National Joint Registry (NJR) for England, Wales and Northern Ireland [7].

### Outcome measurement

All publications were investigated concerning “revision for any reason”. To compare all included studies, which can differ in terms of included cases and investigated time periods, we calculated *revisions per 100 observed component years* (*CY*) which is widely accepted and has been applied repeatedly in the field of orthopaedics [9, 11, 16–18]. Thereupon it is possible to compare different data sources regardless of their follow-up periods and number of UKAs. CY are calculated as number of primary surgeries at follow-up multiplied by mean follow-up time. Considering this calculation, larger cohorts and longer follow-up periods are given higher weight in comparison. The exact principle for the calculation is: number of cases of revision surgery for any reason divided by the number of CY observed and multiplied by 100. A value of one revision per 100 observed CY corresponds to a 1% revision rate at 1 year and a 10% revision rate at 10 years of follow-up. We exclusively included studies with more than 200 CY, as then the denominator is relatively big and more stable against changes in the numerator, respectively, in the number of revision.

### Statistical analysis

Methodologically, we analysed real life data and no “probabilities” and therefore no *p* values could be calculated as previously published [16]. Concerning the significance, we followed the criteria applied in previous investigations [10, 11, 18]. Significance was determined by deviations from the mean by a factor of three (for instance, the revision rates of a dataset are three times as high as in the control group). This generous different factor was applied, as there exist a multitude of potential influence factors among included datasets not related to the prosthesis itself: surgeon’s expertise, patients’ characteristics, surgical techniques, and other circumstances in the particular hospital. Deviations exceeding a threefold difference are not explainable by these confounders and are considered significant. This method is supported by findings from the Swedish and Danish hip arthroplasty register, where the revision rate of every hospital compared to the national mean were within the threefold difference limit. Additionally, single implant mean revision rates do not differ threefold among national registers [10, 16].

## Results

### Dependent and independent UKAs

**Fig. 1:**
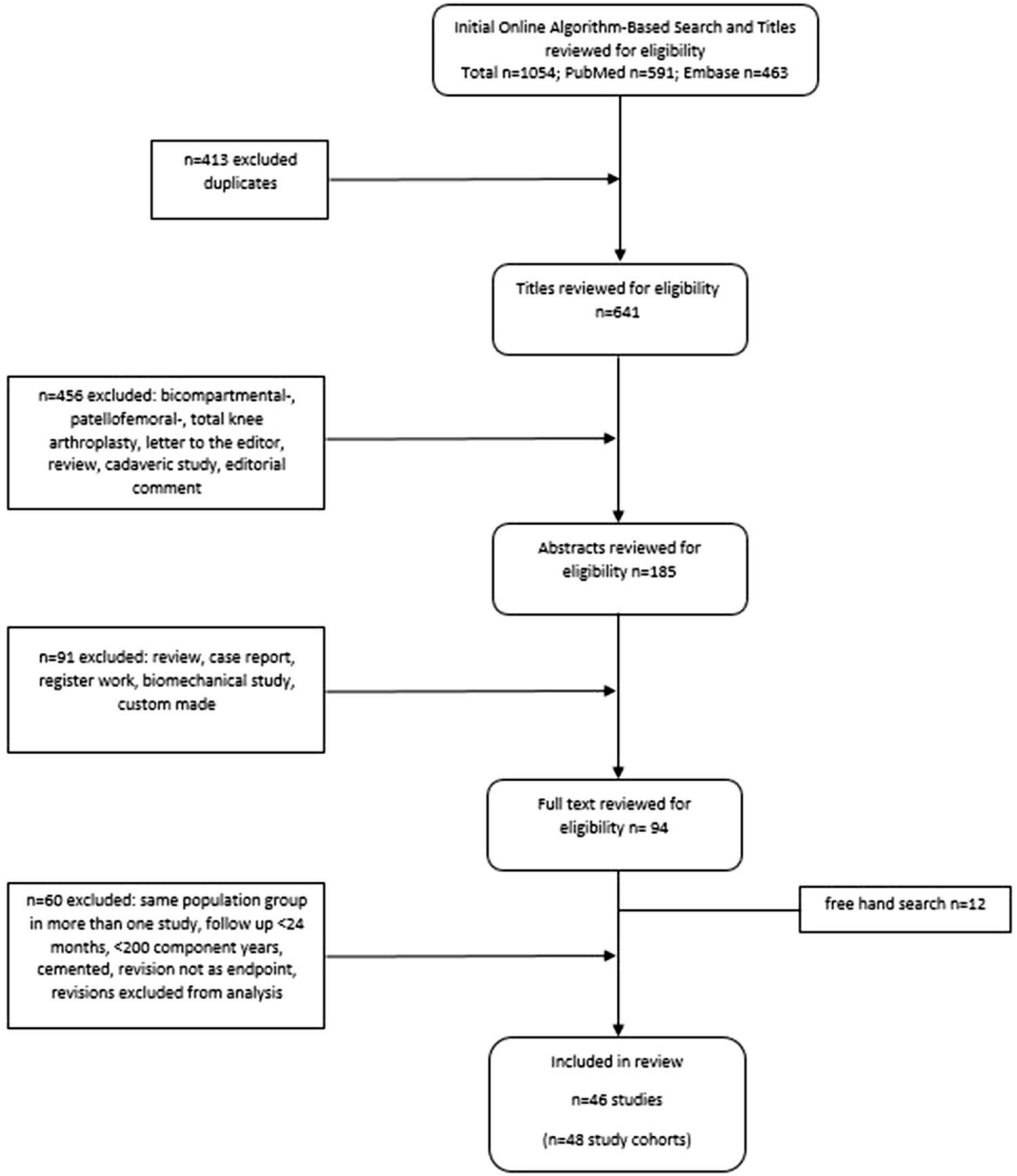
Flow chart of the study identification

In total, 46 studies with 48 study cohorts were revealed through our research and met the inclusion criteria (Table 1). Eleven different implants were used, including prostheses developments from older designs to newer ones within the same company. Oxford UKAs were the most commonly used prostheses among the papers (25/48). 22 investigated groups had more than 1000 CY, indicating short follow-up periods and/or small amounts of primaries in the remaining 26 cohorts. Median follow-up time of all studies was 5.4 years. 12,453 primaries and 791 revisions were assessed. The overall median revision rate per 100 observed CY was 1.11. The flow chart of the study identification is illustrated in Fig. 1 and the detailed results of our evaluation are given in Table 2.

**Table 1:** Included clinical studies with UKA types in the past two decades

Study	UKA	Cases at follow-up	Follow-up (years)	Revisions	Dep/indep
Alnachoukati et al. (2018)	Oxford III	707	7.3	93	Dependent
Tian et al. (2017)	Oxford III	440	6.1	4	Independent
Streit et al. (2017)	Oxford III	112	5.0	5	Dependent
Newman et al. (2017)	Oxford domed lateral	61	7.0	5	Independent
Xue et al. (2017)	Oxford	708	6.2	11	Independent
Seng et al. (2017)	MG/ZUK	106	5.0	0	Independent
Smith et al. (2016)	AMC Uniglide	101	3.9	4	Independent
Bottomley et al. (2016)	Oxford III	1084	5.2	46	Dependent
Forster et al. (2016)	AMC Uniglide	236	7.3	20	Independent
Hawi et al. (2016)	Link Endo Modell	76	8.6	4	Dependent
Koh et al. (2016)	Oxford III	82	2.8	4	Independent
Venkatesh et al. (2016)	MG	175	5.6	7	Independent
Bruni et al. (2016)	Preservation Uni	273	10.2	25	Independent
Baur et al. (2015)	ZUK	132	3.4	5	Independent
Vasso et al. (2015)	ZUK	136	7.5	4	Independent
Pandit et al. (2015)	Oxford III (cemented)	1000	10.3	52	Dependent
Zengerink et al. (2015)	ZUK	137	5.0	18	Independent
Tuncay et al. (2015)	Oxford III	109	3.5	3	Independent
Weston et al. (2014)	Oxford domed lateral	265	4.1	12	Dependent
Burnett et al. (2014)	Oxford III	467	6.1	42	Independent
Iriberri et al. (2014)	Genesis/Accuris	101	5.8	4	Independent
Cepni et al. (2014)	Oxford III	67	5.6	3	Independent
Hamilton et al. (2014)	Preservation Uni	517	4.9	43	Dependent
Cavaignac et al. (2013)	HLS Uni	212	11.6	15	Independent
Schroer et al. (2013)	Oxford III	83	3.6	11	Independent
Altuntas et al. (2013)	Oxford domed lateral	64	3.2	3	Independent
Rachha et al. (2013)	MG	56	10.7	7	Independent
Liebs et al. (2013)	Preservation Uni	558	5.1	44	Dependent
Bergeson et al. (2013)	Oxford III	839	3.7	44	Dependent
Yoshida et al. (2013)	Oxford III	1251	5.2	25	Independent
Heyse et al. (2012)	Genesis/Accuris	223	10.8	15	Dependent
Bhattacharya et al. (2012)	Preservation Uni	91	3.7	6	Independent
Bhattacharya et al. (2012)	Oxford III	49	5.6	1	Independent
Lustig et al. (2012)	HLS Uni	80	5.9	1	Dependent
Lim et al. (2012)	Oxford III	400	5.2	14	Independent
Matharu et al. (2012)	Oxford III	459	4.4	25	Independent
Niinimäki et al. (2011)	Oxford III	113	5.3	20	Dependent
Lisowski et al. (2011)	Oxford III	244	4.2	9	Independent
John et al. (2011)	MG	94	10.8	7	Independent
Pandit et al. (2010)	Oxford III	65	5.0	9	Dependent
Clarius et al. (2010)	Oxford III	61	5.0	2	Independent
Whittaker et al. (2010)	Oxford III	79	3.6	7	Independent
Whittaker et al. (2010)	MG	150	8.1	22	Independent
Biswal et al. (2010)	Allegretto	116	5.7	9	Independent
O’Donnell et al. (2010)	Repicci	114	7.4	22	Independent
Kuipers et al. (2010)	Oxford III	437	2.6	45	Independent
Parratte et al. (2009)	MG	35	9.7	6	Independent
Koskinen et al. (2009)	MG	46	7.0	8	Independent

46 studies with 48 study cohorts were used for further analysis

**Table 2:** Evaluation of unicompartmental knee arthroplasty

	All UKA	All independent UKA	All dependent UKA	Independent study groups Oxford medial UKA	Dependent study groups Oxford medial UKA
Primary implants	12,453	7372	5081	4936	3855
Revisions	791	403	388	206	260
Median follow-up (years)	5.4	5.6	5.0	5.2	7.3
Observed CY	78,086	42,418	35,668	24,837	25,361
Median revisions/100 CY^a^	1.11	1.11	1.00	0.80	1.16
Number of study groups	48	35	13	15	6

Revision rates did not exceed a threefold difference between our observed study groups. None of the investigated parameters of this group analyses showed a significant difference according to the criterion of significance

^a^The median of the revisions/100CY from each study was used for calculation of the overall median revision rate per group

With regard to revisions, there was no significant nor clinically relevant difference between dependent and independent clinical studies. In independent literature, the median rate of revisions for any reason after UKA is 1.11 revisions per 100 observed CY. This corresponds to a revision rate of 11.1% after 10 years and is in line with a calculated 10.0% median revision rate at ten years of follow-up for dependent studies (1.00 revisions/100 CY). Median follow-up between the groups was comparable.

### Medial and lateral UKA

36 studies solely focused on medial UKA with a 0.90 median revision rate per 100 observed CY. Nine studies were rated as dependent and showed a 0.89 median revision rate per 100 observed CY. This is an equal rate compared to 27 independent medial UKA studies (0.90 revisions/100 CY).

Only five studies exclusively comprised lateral UKA of which four implants were developed by the Oxford Group. They showed a median 1.17 revision rate per 100 observed CY, which is slightly higher compared to medial UKA.

### Reason for revision

All studies recorded 791 revisions of primary UKA. The main reasons for revision were loosening (25.8%), progression of arthritis (24.4%) and pain (12.4%). Bearing dislocations (10.2%) were the fourth most commonly mentioned reason as most of the studies used Oxford UKA with a mobile bearing. All details are given in Table 3.

**Table 3:** Primary UKA by reason for revision in clinical studies

Reason for revision	Number	Percent
Loosening	203	25.8
Progression of disease	193	24.4
Pain	98	12.4
Bearing dislocation	81	10.2
Infection	32	4.0
Wear	22	2.8
Lysis	21	2.7
Fracture	20	2.5
Instability	13	1.6
Other	13	1.6
Unknown	95	12.0
Total	791	100

### Oxford III medial UKA

Within this study, we additionally performed an analysis for Oxford III medial UKA. Fifteen independent study groups revealed a median 0.80 revision rate per 100 observed CY. This is a lower rate compared to 1.16 from six dependent studies without reaching our level of significance. The groups did not differ in terms of observed CY, as they were 24,837 and 25,361, respectively (Table 2).

### Registry data of UKA

The evaluation of UKA using worldwide register datasets is illustrated in Table 4 and shows that 10-year revision rates are higher than the average revision rate published in clinical studies. The cumulative percent revision rate (CRR) was estimated using the Kaplan–Meier method in all included registers, whereas the NZJR was the only one to additionally report the outcome in revisions per 100 CY.

**Table 4:** Outcomes from evaluation of arthroplasty registry data

	Primaries	Recording time	Cumulative percent revision rate at 10 years	Observed CY	Rate/100 component years
AOA	49,173	2003–2017	14.7	–	–
NJR	85,312	2003–2017	11.4	–	–
SKAR	6275	2006–2015	14.3	–	–
NZJR	12,627	1999–2018	11.2	86,980	1.20

As regards medial and lateral UKA, the latest AOA report shows that lateral UKA has a higher 10-year rate of revision than medial UKA (14.7% and 13.2%, respectively) [8]. No specific data on revision rates for medial and lateral UKA were found in the other registers.

With regard to Oxford partial knee replacement, arthroplasty registers revealed that Oxford UKAs are the leading implants worldwide. They accounted for 35.0% of all UKAs in the AOA [8], for 56.3% in the NJR [7], for 41.1% in the SKAR [15] and for 69.3% in the NZJR [14]. The 10-year AOA CRR was 14.8% for cemented and 13.5% for cementless Oxford UKA [8]. The latest NJR report showed a 10-year CRR of 11.4% [7].

Further information is illustrated in Table 4.

## Discussion

This report shows that no bias in dependent or developer studies was detected with regard to revision rates of UKA. A fourth of all published clinical studies in this paper were rated as dependent failure rates after 10 years were comparable to independent literature. We estimated a superiority of dependent clinical trials, as they may not be published if they do not meet the study designer’s expectations and are usually conducted by experts in their field. However, this has not been observed and these findings appear to be consistent with results of previous investigators [11, 18].

The amount of implanted prostheses in clinical studies is low compared to register data. All included studies revealed a total of 12,453 implanted UKAs in the past 10 years. These are only twice as many prostheses as are implanted in Sweden during the same period [15]. Observed revision rates in clinical studies were satisfactory after 10 years whereas implant survival in arthroplasty registers was worse. The NJR was the only register that showed almost equal results in comparison to clinical studies [7]. However, these differences did not reach our level of significance. The AOA 10-year cumulative percent revision for UKA is 14.7% compared to 5.3% for TKA [8]. The NJR reveals a 4.3% cumulative percentage probability of a first revision after 10 years for TKA, which is 2.5 lower than UKA [7].

Surprisingly, revision rates of UKA did not improve over a 24‐year follow‐up period according to a report of the Finnish arthroplasty register [19]. On the contrary, survival of TKAs substantially increased during the same period. A possible explanation for this phenomenon could be the use of UKAs over the same follow-up period mainly in low-volume hospitals with less than ten operations per year [20]. These findings are supported by analyses of the Norwegian and Sweden arthroplasty registers: in Norway, the risk of revision was significantly higher for hospitals performing less than 10 Oxford UKA procedures a year than for those performing more than 40 Oxford UKA procedures a year [21]. A Swedish report concluded that there is an association between the number of UKAs performed in a unit and the incidence of subsequent revisions [22]. Within our data we see similar results. If we divide our 48 study cohorts into two similarly sized groups regarding the amount of implanted prostheses, we can calculate a 10-year revision rate of 10% for the group with higher implantation rate (> 117 implantations) versus 12% for the other half with fewer implantations, without significance. Consequently, better results are achieved by more experienced surgeons or high volume units [21, 23]. For comparison, the NJR report states that the number of consultants who entered more than 50 TKA procedures each year was 710 in 2016. For UKA the number of consultants was 34 [7].

There was no difference in this study between medial and lateral UKA in terms of revision rates. The AOA 10 year CRR for primary medial UKA is 14.5% and 15.3% for lateral UKA [8]. In a systematic review, there was also no deviation between 10-year survivorship of medial and lateral UKA with success rates of 91.7% and 91.4% respectively [24].

As most of the UKA developers’ bias controversy originates from conflicting revision rates regarding medial Oxford UKA, we additionally conducted an individual search exclusively on medial Oxford III UKA and their corresponding revision rates. According to Labek et al., the revision rate data of the implant development team in Oxford have been 2.7 times lower than Oxford UKA revision rates from independent literature and 4.4 times lower than arthroplasty registry data [25]. We cannot confirm these findings with our applied criteria. Reasons might be that we did not include publications that were older than 10 years. Moreover, we focused on dependent clinical studies and not only on studies from the inventor group. In this study, publications were also rated as dependent that did not exclusively come from Oxford or one of the inventors, by name Goodfellow or Murray. Dependence also included that the producing company (Biomet Inc.) designed the study or authors were paid consultants by them. We also excluded studies with the same study group in multiple reports and solely used the cohort with the longer follow up period. With respect to registry data, Oxford UKA revision rates were higher compared to clinical studies, but also clearly below a threefold difference.

As natural limitation, meta-analyses are dependent of the quality of the primary data included. Most of the clinical trials are usually conducted in centres of excellence by highly experienced surgeons with great enthusiasm for their implant, whereas registry data consist of many centres and many surgeons for a more heterogeneous patient population. As we have only included publications with more than 200 observed CY this might have caused a possible selection bias. However, an analysis of the revision rate per 100 observed CY is more appropriate with larger cohorts and long-term follow-up [18]. Furthermore, we compared the average revision rate per 100 observed CY from clinical studies with the CRR from arthroplasty registries. CRR uses the Kaplan–Meier method and resembles reality more closely [8]. The calculation of CRR, however, was not possible for clinical studies as there was not enough information for this type of calculation. Nevertheless, results from arthroplasty registries that used both types of calculation showed comparable long-term revision rates [8, 14]. Therefore, the revision rate per 100 observed CY and CRR are suitable for comparison and provide a realistic picture of implant survival.

Most joint replacement registers have used revision rate as the sole measure of outcome, but it is not the only one. Revision rate does not automatically reflect the quality of life and patients’ satisfaction. Some implants show higher frequencies of unsatisfactory outcomes without being revised compared to others [26]. This mismatch applies to UKA as well as TKA, and survival rates might be a misleading outcome measurement in the comparison [23]. Within our data, we could not perform any solid analysis with functional outcome scores as given scores were too heterogeneous and observation periods too different. Next, we cannot state any recommendations in terms of surgical approaches, implant and bearing types, or fixation techniques.

## Conclusion

The outcomes of UKA in dependent and independent clinical studies do not differ significantly and are in line with arthroplasty register datasets. We cannot confirm biased results and the authors recommend the use of UKAs in properly selected patients by experts in their field.

## References

[1] Hauer G, Sadoghi P, Bernhardt GA, Wolf M, Ruckenstuhl P, Fink A (2019). Greater activity, better range of motion and higher quality of life following unicompartmental knee arthroplasty: a comparative case–control study. Arch Orthop Trauma Surg.

[2] Argenson JN, Parratte S (2006). The unicompartmental knee: design and technical considerations in minimizing wear. Clin Orthop Relat Res.

[3] Hamilton WG, Ammeen DJ, Hopper RH (2014). Mid-term survivorship of minimally invasive unicompartmental arthroplasty with a fixed-bearing implant: revision rate and mechanisms of failure. J Arthroplast.

[4] Koh IJ, Kim JH, Jang SW, Kim MS, Kim C, In Y (2016). Are the Oxford((R)) medial unicompartmental knee arthroplasty new instruments reducing the bearing dislocation risk while improving components relationships? A case control study. Orthop Traumatol Surg Res.

[5] W-Dahl A, Robertsson O, Lidgren L, Miller L, Davidson D, Graves S (2010). Unicompartmental knee arthroplasty in patients aged less than 65. Acta Orthop.

[6] Liddle AD, Judge A, Pandit H, Murray DW (2014). Adverse outcomes after total and unicompartmental knee replacement in 101,330 matched patients: a study of data from the National Joint Registry for England and Wales. Lancet.

[7] National Joint Registry for England and Wales (2018). National Joint Registry. 15th Annual Report 2018. National Joint Registry for England and Wales. Available at: http://www.njrcentre.org.uk. Accessed 27 Nov 2019

[8] Australian Orthopaedic Association National Joint Replacement Registry (2018). National Joint Replacement Registry. Annual Report 2018. Australian Orthopaedic Association National Joint Replacement Registry. https://aoanjrr.sahmri.com/annual-reports-2018. Accessed 27 Nov 2019

[9] Sadoghi P, Janda W, Agreiter M, Rauf R, Leithner A, Labek G (2013). Pooled outcome of total hip arthroplasty with the CementLess Spotorno (CLS) system: a comparative analysis of clinical studies and worldwide arthroplasty register data. Int Orthop.

[10] Quality of publications regarding the outcome of revision rate after arthroplasty. Final Report of the QoLA Project. Presented at the EFORT Congress 2011 in Copenhagen. http://www.ear.efort.org/downloads/E-book_QoLA%20Project_Final%20Report_EFORT%20Copenhagen. Accessed 27 Nov 2019

[11] Pabinger C, Berghold A, Boehler N, Labek G (2013). Revision rates after knee replacement. Cumulative results from worldwide clinical studies versus joint registers. Osteoarthr Cartil.

[12] Moher D, Liberati A, Tetzlaff J, Altman DG, PRISMA Group (2009). Preferred reporting items for systematic reviews and meta-analyses: the PRISMA Statement. Open Med.

[13] (2016) EFORT Website for European Arthroplasty Registers, EAR Welcome. http://www.ear.efort.org. Accessed 27 Nov 2019

[14] New Zealand National Joint Register (2018). The New Zealand Joint Register. Nineteen Year Report. New Zealand National Joint Register. http://www.cdhb.govt.nz/njr. Accessed 27 Nov 2019

[15] Swedish Knee Arthroplasty Register (2017). Annual Report 2017. http://www.myknee.se/pdf/SVK_2017_Eng_1.0.pdf. Accessed 27 Nov 2019

[16] Pabinger C, Bridgens A, Berghold A, Wurzer P, Boehler N, Labek G (2015). Quality of outcome data in total hip arthroplasty: comparison of registry data and worldwide non-registry studies from 5 decades. Hip Int.

[17] van der List JP, Chawla H, Zuiderbaan HA, Pearle AD (2017). Survivorship and functional outcomes of patellofemoral arthroplasty: a systematic review. Knee Surg Sports Traumatol Arthrosc.

[18] Hauer G, Vielgut I, Amerstorfer F, Maurer-Ertl W, Leithner A, Sadoghi P (2018). Survival rate of short-stem hip prostheses: a comparative analysis of clinical studies and National Arthroplasty Registers. J Arthroplast.

[19] Koskinen E, Eskelinen A, Paavolainen P, Pulkkinen P, Remes V (2008). Comparison of survival and cost-effectiveness between unicondylar arthroplasty and total knee arthroplasty in patients with primary osteoarthritis: a follow-up study of 50,493 knee replacements from the Finnish Arthroplasty Register. Acta Orthop.

[20] Koskinen E, Paavolainen P, Eskelinen A, Pulkkinen P, Remes V (2007). Unicondylar knee replacement for primary osteoarthritis: a prospective follow-up study of 1,819 patients from the Finnish Arthroplasty Register. Acta Orthop.

[21] Badawy M, Espehaug B, Indrekvam K, Havelin LI, Furnes O (2014). Higher revision risk for unicompartmental knee arthroplasty in low-volume hospitals. Acta Orthop.

[22] Robertsson O, Knutson K, Lewold S, Lidgren L (2001). The routine of surgical management reduces failure after unicompartmental knee arthroplasty. J Bone Joint Surg Br.

[23] Goodfellow JW, O’Connor JJ, Murray DW (2010). A critique of revision rate as an outcome measure: re-interpretation of knee joint registry data. J Bone Joint Surg Br.

[24] van der List JP, McDonald LS, Pearle AD (2015). Systematic review of medial versus lateral survivorship in unicompartmental knee arthroplasty. Knee.

[25] Labek G, Sekyra K, Pawelka W, Janda W, Stockl B (2011). Outcome and reproducibility of data concerning the Oxford unicompartmental knee arthroplasty: a structured literature review including arthroplasty registry data. Acta Orthop.

[26] Labek G, Thaler M, Janda W, Agreiter M, Stockl B (2011). Revision rates after total joint replacement: cumulative results from worldwide joint register datasets. J Bone Joint Surg Br.

